# Assessment of left atrial mechanical functions and atrial electromechanical delay in Juvenile idiopathic arthritis by tissue Doppler echocardiography

**DOI:** 10.1186/s12969-016-0122-4

**Published:** 2016-11-24

**Authors:** Azza Z. El Eraky, Nesrin M. Handoka, Mona Sayed Ghaly, Samah Ismail Nasef, Nahed A. Eldahshan, Ahmed M. Ibrahim, Sherein Shalaby

**Affiliations:** 1 Department of Cardiology, Faculty of Medicine, Portsaid University, Portsaid, Egypt; 2 Department of Pediatrics, Faculty of Medicine, Portsaid University, Portsaid, Egypt; 3 Department of Physical Medicine, Rheumatology and Rehabilitation, Faculty of Medicine, Suez Canal University, Ismailia, Egypt; 4 Department of Family Medicine, Faculty of Medicine, Suez Canal University, Ismailia, Egypt; 5 Department of Pediatrics, Faculty of Medicine, Suez Canal University, Ismailia, Egypt

**Keywords:** Juvenile idiopathic arthritis, Echocardiography, Doppler, Ventricular dysfunction, Atrial mechanical function

## Abstract

**Background:**

Juvenile idiopathic arthritis (JIA) is a systemic chronic inflammatory disease. Studies using tissue Doppler imaging (TDI) for the evaluation of cardiac functions of children with JIA are limited. Thus, this study was conducted to evaluate Left ventricular function, left atrial mechanical functions and atrial electromechanical delay in JIA.

**Methods:**

This study was carried out as a across sectional study. A total of 34 patients with active JIA and 34 controls were included. Atrial electromechanical delay and left atrial (LA) mechanical functions in addition to systolic and diastolic left ventricular (LV) functions were measured by using conventional echocardiography and TDI. Assessment of disease activity was done using Juvenile arthritis disease activity score (JADAS-27).

**Results:**

JIA patients had abnormal atrial electromechanical coupling as established from prolonged lateral mitral annulus (PA lateral), septal mitral annulus (PA septum), inter-atrial and intra-atrial electromechanical delays compared with healthy controls. Left ventricular filling abnormalities were found characterized by a reduced E/A ratio (1.07 ± 0.56 *vs.* 1.48 ± 0.16, *p* = 0.01). E/Em was significantly higher in patients with JIA (7.58 ± 1.79 *vs.* 4.74 ± 1.45, *p* = 0.003) denoting impaired diastolic function. Left atrial mechanical functions assessment showed significantly decreased LA passive emptying fraction, increased LA active emptying fraction and LA total emptying volume in JIA patients (*p* = 0.01, *p* = 0.01, *p* = 0.03 respectively).

**Conclusion:**

Atrial electromechanical coupling intervals, and LA mechanical functions were impaired which can be considered as an early form of subclinical cardiac involvement in JIA patients. Significant diastolic functional abnormalities exist in JIA.

## Background

Juvenile idiopathic arthritis (JIA) is defined as ‘persistent arthritis of unknown etiology that begins earlier than 16 years and persists for at least 6 weeks’ [1]. It affects about 1 in 1,000 children worldwide and is the most common cause of autoimmune musculoskeletal disorders [2]. It represents up to 65% of arthritic diseases in children and is one of the highest five chronic illnesses in children [3]. Cardiac (pericardial, myocardial or endocardial) involvements in JIA are usually subclinical. However, the long-term risk, the clinical course and prognosis of cardiac involvement in JIA is unclear [4]. Rhythm and conduction disturbances when manifested are considered as a significant cause of cardiovascular (CV) morbidity and mortality in patients with JIA [5]. Atrial fibrillation (AF) is the most common arrhythmia of clinical significance [6].

Nowadays, it has been recognized that inflammation plays a significant role in the pathogenesis of atrial fibrillation (AF) [7]. The risk of AF is increased in patients with rheumatic diseases such as rheumatoid arthritis, systemic lupus erythematosus and psoriasis, which all implicate systemic inflammation in their pathogenesis [8]. However, the relationship between JIA and AF is not yet obviously understood as in other rheumatic diseases.

Electrophysiological and electromechanical abnormalities originating from conduction disturbances in the atria are associated with an increased risk of AF [9, 10]. The prolongation of intra- and interatrial electromechanical delay and the non-uniform transmission of sinus impulses are frequent electrophysiological features of the atria predisposed to fibrillation [11]. Atrial electromechanical delay may be measured using invasive and noninvasive techniques. With recent developments in echocardiographic techniques, Tissue Doppler imaging (TDI) has become an alternative noninvasive method [9].

Tissue Doppler imaging (TDI) has been proved to provide atrial conduction times, measurements of local systolic and diastolic myocardial velocities as well as detection of abnormalities of left ventricular diastolic function [12, 13]. An increase in atrial electromechanical delay duration measured with this method was shown to be an independent variable for AF development [9].

To the extent of our knowledge, the published studies assessed left ventricular diastolic dysfunction by TDI without assessing atrial electromechanical delay and LA mechanical functions in JIA patients [14]. Therefore, this study aimed at assessing atrial electromechanical coupling, LA mechanical functions and subclinical LV systolic and diastolic functions using tissue Doppler and correlating atrial electromechanical delay to LA mechanical functions, LV functions and disease activity in Suez Canal area JIA patients.

## Methods

This study was carried out as a cross sectional study. Thirty-four active JIA patients with a mean age of 13.97 ± 4.77 years, fulfilling the revised criteria of the International League of Associations for Rheumatology (ILAR) [15] were enrolled for assessment at the Pediatric, Cardiology and Physical Medicine, Rheumatology and Rehabilitation departments as well as Family Medicine Centers and Clinics affiliated to Faculty of Medicine, Suez Canal University in Ismailia, Suez and Port-Said governorates. In addition, age-gender matched 34 healthy children attending the outpatients’ clinics for non-medical causes were randomly recruited to the Study as control.

All study Children older than 16 years; children with rheumatic or congenital heart disease, history of any clinical evidence of coexisting cardiac disease, diabetes mellitus, arrhythmia, valvular heart diseases or ischemic heart diseases were excluded by history, physical examination, chest radiography, Echocardiography and standard 12-lead electrocardiography (ECG).

Prior to subject recruitment, the study protocol was reviewed and approved by the research ethics committee of the Faculty of Medicine, Suez Canal University, written informed consents were obtained from all the Parents or care givers. All procedures performed in studies involving human participants were in accordance with the ethical standards of the research ethics committee of the Faculty of Medicine Suez Canal University and with the 1964 Helsinki declaration and its later amendments.

We gathered information regarding patient characteristics such as age, gender and disease duration. Growth parameters measured were height (to nearest 0.5 cm via Harpenden stadiometer), weight (to nearest 0.1 kg), and body mass index (BMI) was calculated as weight (kg)/height (m)^2^ [16]. All subjects were managed according to the American College of Rheumatology (ACR) guidelines for the medical therapy of children with Juvenile Idiopathic Arthritis [17].

### Juvenile arthritis disease activity score (JADAS-27) calculation

The JADAS-27 is calculated by summing the scores of four core-set criteria [18]: 1) physician global rating of overall disease activity, measured on a 10-cm horizontal Visual Analog Scale (VAS) (0 = no activity; 10 = maximum activity for both VAS); 2) parent/child ratings of well-being and pain, assessed on a 10-cm horizontal VAS (0 = best; 10 = worst for both VAS); 3) number of active joints, defined as joint swelling or limitation of movement accompanied by pain and tenderness, assessed in 27 joints; and 4) Westergren Erythrocyte Sedimentation Rate (ESR). JADAS is scored on a continuous scale from 0–57. JADAS-27 cut-off score is ≤2.7 for low disease activity and ≥6 for high disease activity [19, 20].

#### Transthoracic Echocardiographic (TTE) study

Transthoracic echocardiographic measurements were performed with General Electric system VIVED 7 machine ultrasound phased array system using a 3.0-mHz transducer for two-dimensional M-mode measurements of the left ventricular structure and systolic function according to the recommendations of the American Society of Echocardiography [21].

Measurements were recorded as average of three cardiac cycles. Left atrium (LA) dimension, left ventricular end-systolic (LVESD) and end-diastolic dimensions (LVEDD), diastolic ventricular septal thickness and diastolic LV posterior wall thickness were measured in the parasternal long-axis view. Left ventricular EF was estimated using the Simpson’s rule. Left ventricular mass was calculated with the Devereux equation [22] and was indexed to body surface area (BSA).

Left ventricular diastolic function was assessed by measuring the mitral flow velocity recorded in the apical four-chamber view. The pulse Doppler sample volume was placed in the left ventricular inflow tract at the level of mitral leaflet tips and three consecutive measurements were averaged. The various variables of diastolic function that were measured included: peak early (E; m/sec) and peak atrial filling velocity (A; m/s), ratio of E to A (E/A), E acceleration time, E deceleration time (Edt; m/s), isovolumic relaxation time (IVRT) was measured in the apical five chamber view with the sample volume placed between the aorta and mitral valve where the recordings of both valves were taken simultaneously.

#### Pulsed wave-tissue Doppler measurements

Tissue Doppler echocardiography was performed with a concurrent Electrocardiography (ECG) recording using Fukuda Denshi FCP-7101 Interpretive ECG Machine. The frequency of the transducer was of 3.5–4.0 MHz, adjusting the spectral pulsed Doppler signal filters to obtain the Nyquist limit of 15–20 cm/sec, and using the minimal optimal gain setting. The monitor sweep speed was set at 50–100 mm/sec to optimize the spectral display of myocardial velocities. A sample volume (2 mm) was placed within mitral annulus septal myocardial wall, analyzing wall motion parallel to cursor orientation. Septal annulus now is the preferred site for tissue Doppler imaging, because this location is less influenced by the pericardium. Systolic (Sm), early diastolic (Em) and late diastolic (Am) TD velocities were measured, and the Em/Am ratio and the dimensionless parameter E/Em were computed. E/Em was found in previous studies to be directly correlated with impaired diastolic function [23–25].

#### Atrial electromechanical coupling

In apical four-chamber view, the pulsed Doppler sample volume was placed at the level of the LV lateral mitral annulus, and subsequently at the septal mitral annulus and right ventricular tricuspid annulus. The sampling window was positioned as parallel as possible with the myocardial segment of interest to obtain the optimal angle of imaging. Atrial electromechanical coupling was defined as the time interval from the onset of P wave on the surface electrocardiogram to the beginning of the late diastolic wave (Am wave), i.e. PA interval and was measured from the lateral mitral annulus (PA lateral), septal mitral annulus (PA septum), and right ventricular tricuspid annulus (PA tricuspid) [9]. All PA intervals were averaged over three consecutive beats. The difference between the lateral and tricuspid PA intervals was defined as interatrial electromechanical delay, and the difference between the septal and tricuspid PA intervals was defined as intra-atrial electromechanical delay [9].

$$ \mathrm{P}\mathrm{A}\ \mathrm{lateral}\hbox{-} \mathrm{P}\mathrm{A}\ \mathrm{tricuspid}\left(\mathrm{msec}\right)=\mathrm{inter}\hbox{-} \mathrm{atrial}\ \mathrm{electromechanical}\ \mathrm{delay}, $$

$$ \mathrm{P}\mathrm{A}\ \mathrm{septum}\hbox{-} \mathrm{P}\mathrm{A}\ \mathrm{tricuspid}\left(\mathrm{msec}\right) = \mathrm{intra}\hbox{-} \mathrm{atrial}\ \mathrm{electromechanical}\ \mathrm{delay}. $$

#### Assessment of left atrial mechanical functions

Left atrial volumes were measured by the method of discs in the apical four-chamber view at end-systole (maximal, Vmax), end-diastole (minimal, Vmin) and at the onset of atrial systole (P wave on electrocardiogram, Vp). All volumes were indexed to BSA and expressed in ml/m2. Then, the following LA emptying function parameters were calculated [22]:

$$ \mathrm{L}\mathrm{A}\ \mathrm{p}\mathrm{assive}\ \mathrm{emptying}\ \mathrm{volume}=\mathrm{Vmax}\hbox{-} \mathrm{V}\mathrm{p}; $$

$$ \mathrm{L}\mathrm{A}\ \mathrm{p}\mathrm{assive}\ \mathrm{emptying}\ \mathrm{fraction}=\left(\mathrm{Vmax}\hbox{-} \mathrm{V}\mathrm{p}\right)/\mathrm{Vmax}; $$

$$ \mathrm{L}\mathrm{A}\ \mathrm{active}\ \mathrm{emptying}\ \mathrm{volume}=\mathrm{V}\mathrm{p}\hbox{-} \mathrm{Vmin}; $$

$$ \mathrm{L}\mathrm{A}\ \mathrm{active}\ \mathrm{emptying}\ \mathrm{fraction}=\left(\mathrm{V}\mathrm{p}\hbox{-} \mathrm{Vmin}\right)/\mathrm{V}\mathrm{p}; $$

$$ \mathrm{Conduit}\ \mathrm{volume}=\left[\mathrm{L}\mathrm{V}\ \mathrm{stroke}\ \mathrm{volume}\hbox{-} \left(\mathrm{Vmax}\hbox{-} \mathrm{Vmin}\right)\right];\ \mathrm{and}\ \mathrm{L}\mathrm{A}\ \mathrm{total}\ \mathrm{emptying}\ \mathrm{volume}=\mathrm{Vmax}\hbox{-} \mathrm{Vmin}. $$

### Statistical analysis

All statistical analyses were performed using the IBM SPSS for Windows version 22.0 (SPSS Armonk, NY: IBM Corp USA). Data were expressed as mean and standard deviation. Student’s t test (two-tailed) was used for inter-group comparisons. To analyze categorical data, chi-square test was used. Nonparametric data were analyzed using Mann Whitney U test. Pearson correlation coefficient test was used to assess the degree of association between different variables. Pearson correlation was used to correlate continuous variables. *P* values were adjusted using false discovery rate (FDR) algorithm. Multivariate linear regression analysis was used to identify significant determinants of interatrial and intra-atrial electromechanical delay. A *p* value of less than 0.05 was considered significant.

## Results

Clinical and laboratory characteristics of the two groups are shown in Table 1. Owing to the fact that the most prevailing presentation was the poly-articular JIA (91.2%) and to avoid outlier we excluded the results of patients with systemic and oligo-articular JIA (3 patients) from the analyses. Age, sex, body mass index, systolic and diastolic blood pressure, and heart rate, were similar between the two groups (*p* > 0.05).

**Table 1:** Clinical and laboratory characteristics of the study population

Variables	Patients with JIA (*n* = 34)^a^	Control group (*n* = 34)	*P* value**
Age (years)	13.75 ± 4.36	12.55 ± 3.54	0.89
Gender			
[Female, *n* (%)]	28(82.4%)	24(70.6%)	0.72
[Male, *n* (%)]	6(17.6%)	10 (29.4%)	
Classification		-	
[Systemic, *n*(%)]	2 (6%)		
[Polyarticular, *n*(%)]	31 (91.2%)		
[Oligoarticular, *n*(%)]	1 (2.9%)		
[Psoriatic, *n*(%)]	1 (2.9%)		
[Enthesitis related, *n*(%)]	0		
[Unspecified, *n*(%)]	0		
Body mass index (kg/m2)	18.56 ± 4.8	17.35 ± 2.09	0.82
Systolic blood pressure (mmHg)	103.87 ± 13.58	93.21 ± 4.18	0.72
Diastolic blood pressure (mmHg)	65.81 ± 7.56	65.06 ± 5.46	0.84
Heart rate (beats/min)	95.9 ± 13.06	84.12 ± 13.24	0.82
Erythrocyte sedimentation rate (mm/hr.)	32.74 ± 11.55	12.68 ± 2.34	0.01*
C-reactive protein (mg/l)	6.72 ± 2.43	3.12 ± 1.94	0.03*
Rheumatoid factor (+ve)	3 (8.8%)	0 (0%)	
JADAS-27	23.51 ± 9.53	-	
Disease duration (months)	50.24 ± 15.98	-	
Medications:		-	
Patients currently treated with Corticosteroid	4 (11.8%)		
Patients previously treated with Corticosteroid	9 (26.5%)		
Patients treated with NSAIDs	26 (76.5%)		
Patients treated with methotrexate	31 (91.2%)		
Patients treated with Biologic (Etanercept)	3 (8.8%)		

*RF* rheumatoid factor, −*ve* negative, *+ve* positive, *JADAS-27* Juvenile arthritis disease activity score, *NSAIDs* non- steroidal anti-inflammatory drugs

*Statistically significant at *p* < 0.05 using Mann-Whitney U test; **p < 0.05 adjusted by false discovery rate (FDR) (Benjamini-Hochberg)

^a^all statistical analyses were performed for 31 patients with polyarticular JIA after exclusion of oligo-articular and systemic JIA patients

Patients with JIA exhibited significantly higher levels of ESR and C reactive protein (CRP). All patients exhibited active disease, the mean JADAS-27 score was 23.51 ± 9.53 and the mean disease duration was 50.24 ± 15.98 months. Methotrexate was the mostly used disease modifying anti-rheumatic drugs (DMARDs) (91.2%) in treating JIA patients.

The main echocardiographic and Doppler findings in this series of patients with JIA without clinical evidence of cardiovascular disease were summarized in Table 2. LV end-diastolic dimension (LVEDD), stroke volume, LV mass index, LA dimension, ejection fraction (EF), fractional shortening (FS) and Peak A velocity were comparable between the two groups. Patients with JIA showed a higher left ventricular end systolic diameter (LVESD) (*P* = 0.01). Peak trans-mitral E velocity was lower among patients with JIA. This was also echoed by the E/A velocity ratio, which was significantly lower among patients compared to the healthy population. E/Em was significantly higher in patients with JIA than in the control group (*p* = 0.02).

**Table 2:** Echocardiographic characteristics of the study population

Variables	Patients with JIA (*n* = 31)^a^	Control group (*n* = 34)	*p* value*
LV End-diastolic dimension (mm)	41.77 ± 5.07	40.33 ± 1.83	0.27
LV End-systolic dimension (mm)	27.97 ± 4.65	24.03 ± 2.97	0.01*
LV End-diastolic volume (cm3)	118.48 ± 23.224	80.44 ± 22.56	0.01*
LV End-systolic volume (cm3)	67.06 ± 6.47	35.91 ± 13.87	0.01*
Stroke volume (mL)	49.10 ± 12.79	52.42 ± 21.33	0.65
Cardiac output (L/min)	5.9 ± 8.5	5.4 ± 10.07	0.03*
Fractional shortening (%)	33.19 ± 4.09	41.03 ± 5.241	0.01*
Ejection fraction (%)	62.26 ± 5.60	71.70 ± 6.152	0.01*
LV Mass index (g/m2)	70.42 ± 12.26	71.70 ± 6.15	0.39
Septum thickness (mm)	7.22 ± 1.54	7.15 ± 0.834	0.94
Posterior wall thickness (mm)	7.30 ± 1.16	7.39 ± 1.14	0.92
Left atrium dimension (mm)	27.97 ± 4.65	26.82 ± 4.87	0.73
E (cm/s)	0.795 ± .0.16	0.99 ± 0.23	0.03*
A (cm/s)	0.64 ± 0.31	0.66 ± 0.21	0.6
E/A ratio	1.07 ± 0.56	1.48 ± 0.16	0.01*
Mitral deceleration time (ms)	150.01 ± 62.54	149.30 ± 107.54	0.94
Isovolumic relaxation time (ms)	72.91 ± 25.4	72.88 ± 9.58	0.94
LV Sm (cm/s)	0.09 ± 0.02	0.08 ± 0.02	0.36
LV Em (cm/s)	0.18 ± 0.06	0.17 ± 0.03	0.19
LV Am (cm/s)	0.19 ± 0.47	0.07 ± 0.02	0.03*
E/Em	7.58 ± 1.79	4.74 ± 1.45	0.01*
Em/Am	1.47 ± 0.64	1.87 ± 0.21	0.02*
LV MPI	69.42 ± 13.41	71.70 ± 6.152	0.47

*p < 0.05 adjusted by false discovery rate (FDR) (Benjamini-Hochberg)

^a^systemic onset and oligo-articular Subtypes were excluded from the analyses

### Left atrial mechanical functions

Left atrial volume measurements (Table 3). The two groups were similar with respect to maximum LA volume, Vp, Vmin, LA passive emptying volume and LA active emptying volume and conduit volume (*p* > 0.05). However, LA passive emptying fraction was significantly decreased and LA active emptying fraction and LA total emptying volume were significantly increased in JIA patients (*p* = 0.01, *p* = 0.01, *p* = 0.03 respectively).

**Table 3:** Left atrial volume measurements of the study population

Characteristics	Patients with JIA (*n* = 31)	Control group (*n* = 34)	*P*-value*
Maximum volume at end-systole (ml/m2)	28.74 ± 11.17	27.85 ± 9.8	0.51
Volume at the beginning of atrial systole (ml/m2)	18.71 ± 7.63	18.15 ± 7.36	0.65
Minimal volume at end-diastole (ml/m2)	11.19 ± 7.61	11.3 ± 5.39	0.27
Passive emptying volume (ml/m2)	10.03 ± 4.20	9.70 ± 3.64	0.6
Passive emptying fraction (%)	23.66 ± 9.98	35.56 ± 8.86	0.01*
Conduit volume (ml/m2)	31.55 ± 11.54	35.88 ± 22.37	0.43
Active emptying volume (ml/m2)	7.52 ± 3.29	6.85 ± 3.43	0.65
Active emptying fraction (%)	41.13 ± 11.54	32.37 ± 7.13	0.01*
Total emptying volume (ml/m2)	18.04 ± 6.07	16.55 ± 5.49	0.03*

*p < 0.05 adjusted by false discovery rate (FDR) (Benjamini-Hochberg)

### Atrial electromechanical coupling

Atrial electromechanical coupling intervals were measured from different areas by TDI (Table 4). JIA patients had significantly prolonged PA lateral (*p* = 0.03), PA septum (*p* = 0.02), inter-atrial (*p* = 0.04) and intra-atrial (*p* = 0.02) electromechanical delays compared with healthy controls.

**Table 4:** Atrial electromechanical coupling findings measured by tissue Doppler imaging

Variables	Patients with JIA (*n* = 34)	Control group (*n* = 34)	*p* value*
PA lateral (msec)	56.58 ± 19.79	48.27 ± 11.31	0.03*
PA septum (msec)	48.68 ± 25.03	39.06 ± 18.39	0.02*
PA tricuspid (msec)	31.77 ± 12.53	28.18 ± 12.05	0.08
PA lateral - PA tricuspid (msec)^a^	23.52 ± 12.04	19.01 ± 10.486	0.04*
PA septum - PA tricuspid (msec)^b^	17.02 ± 15.24	10.88 ± 13.610	0.02*

*PA* the interval with tissue Doppler imaging, from the onset of P wave on the surface electrocardiogram to the beginning of the late diastolic wave (Am wave)

*p < 0.05 adjusted by false discovery rate (FDR) (Benjamini-Hochberg)

^a^Interatrial electromechanical delays

^b^intra-atrial electromechanical delays

Multivariate linear regression analysis showed that Inter-atrial electromechanical delay was significantly related with disease duration (*p* = 0.025), JADAS-27 (*p* = 0.045) and Systolic Blood pressure (*p* = 0.013) (adjusted *R* ^*2*^ = 0.16). Intra-atrial electromechanical delay was related to disease duration (*p* = 0.003) and JADAS-27 (*p* = 0.01) (adjusted *R* ^*2*^ = 0.39). No association was found between Intra-atrial electromechanical delay and LA mechanical functions and to LV functions in these patients.

## Discussion

Solid data concerning cardiovascular risk in JIA patients is still deficient [4]. However, evidence showed that subclinical CV involvement in JIA can starts just after the onset of the disease and exacerbates during the course of the disease. All cardiac structures may be involved with variable complications [5].

The present study showed that there was no significant difference between the JIA patients and age and sex-matched control group in terms of heart rate, systolic and diastolic blood pressures.

Heart rate proved to be elevated within normal limits in many studies [26–28], only one investigation showed significant elevation of heart rate [29]. This elevation could be linked to the inflammatory characteristic of the disease and the associated released mediators [28, 29].

Systolic and diastolic blood pressures results were in accordance with Oguz et al. [26], while studying 30 children and young subjects with JIA (aged 3–15 years), and compared echocardiographic findings of the same number of age and sex-matched controls found that systolic and diastolic pressures were higher in the JIA group although values remained within normal values. Similarly, other researches confirmed the same findings [28, 29]. These results were previously attributed to the use of steroids and nonsteroidal anti-inflammatory drugs in therapy, thus leading to salt and water retention [28]. However, this issue has been closely considered by Breda et al. [30] who studied potential atherosclerotic changes in 38 children with oligo- and polyarticular JIA. Blood pressure was initially assessed revealing significantly higher systolic and diastolic blood pressure in JIA group when compared with controls. After 12 months a significant reduction in blood pressure was observed. They suggested that this change may be due to better disease control.

Thus, patients with JIA need close monitoring of blood pressure during follow up for early detection of potential hypertension and adequate treatment [26–29].

Conduction system disorders, especially atrial fibrillation (AF), are evident in patients with rheumatic disorders compared to healthy population as reported from literature [31]. In our study, atrial electromechanical delay was evaluated by means of tissue Doppler echocardiography with simultaneous ECG recording. We found that JIA patients had significantly prolonged PA lateral, inter-atrial and intra-atrial electromechanical delays compared with healthy controls. These abnormalities are usually associated with a higher risk for AF [11].

On the contrary, one research studied atrial conduction abnormalities through measuring P wave dispersion (PWD) and P wave duration and found that PWD value was similar in JIA patients and healthy children [32]. They suggested that in relation of PWD patients with JIA are not prone to atrial arrhythmias. We attribute this variation in results to the different methodology used in our study as we used TDI with simultaneous ECG recording instead of the standard ECG alone.

Our results revealed significant increase in LVESD. Systolic function parameters (EF and FS) were lower compared to controls but within normal limits. Several studies were in harmony with our results, where they reported significant higher LVESD and LVEDD [29] or higher LVESD and lower EF compared to controls [26]. Additionally, Koca et al. [27], did not demonstrate any significant differences in the LVESD, LVEDD, EF and FS. Even though within normal range, increased resting heart rate and diastolic LV dimensions together with decreased EF and FS in JIA patients compared to healthy controls, may be an indicator of subclinical myocarditis [14].

By analyzing the measurements of the Pulsed Wave-Tissue Doppler, they revealed abnormalities in diastolic function. Peak trans-mitral E velocity and E/A velocity ratio was significantly lower among JIA patients compared to the healthy group which supports diastolic dysfunction. Our data are consistent with the data of many studies [14, 28, 29, 33].

Concerning TDI, our results showed that E/Em, was significantly higher and Em/Am ratio was significantly lower in JIA patients than in control group. Alkady et al. [28] have observed that the mitral A wave and the isovolumic relaxation time were higher compared to control group, while the mitral E wave and the E/A ratio were found to be lower. Moreover, other study found that, among TDI parameters, mitral annular early diastolic velocity (Em), Em/Am ratio, E wave velocity trace integral (E VTI) and A velocity trace integral (A VTI) were found to be lower in active JIA patients than in the control group which support diastolic dysfunctions in active JIA patients [14].

Diastolic dysfunction is associated with long disease duration, but its etiology is still unclear [34]. Some researchers had explained that these abnormalities could hypothetically be due to an increase in afterload, a decrease in preload or weakened relaxation of the left ventricle, probably due to a myocardial fibrotic process [27]. However, diastolic dysfunction may be also related to a transient myositis or other inflammatory disease manifestation and may have no bearing on future cardiovascular risk [4]. Left atrial mechanical function is an important determinant of LV filling. Left atrial mechanical functions consist of several functions at different stages of cardiac cycle including reservoir, channel and booster pump functions. In case of LV dysfunction, the left atrium could maintain sufficient cardiac output by regulation of these functions [35].

In our work, we found that, LA mechanical functions were significantly impaired as LA passive emptying fraction was significantly decreased while LA active emptying fraction and LA total emptying volume were significantly increased in JIA patients.

To our knowledge, LA mechanical functions was not assessed in JIA. However, other studies have reported LA impaired mechanical functions measured with TDI in other rheumatic disease, as in patients with asymptomatic scleroderma [36].

Furthermore, in order to achieve our aim, atrial electromechanical delay (AEMD) was correlated to LA mechanical functions, LV functions and disease activity in JIA patients. Multivariate linear regression analysis showed that Inter-atrial electromechanical delay was significantly correlated with disease duration, JADAS-27 and Systolic Blood pressure but this correlation showed weak linear relationship (adjusted *R* ^*2*^ = 0.16). Intra-atrial electromechanical delay showed significant correlation to disease duration and JADAS-27 and this correlation showed strong linear relationship (adjusted *R* ^*2*^ = 0.39). These results indicated that cardiac damage is most probably caused by long-term chronic inflammation and high disease activity. Similarly, results of a study carried out on adult RA patients, suggested that intra- and interatrial electromechanical delays are prolonged in RA patients, and that LA active emptying fraction and serum CRP level are independent factors of the inter-atrial electromechanical delay [11].

Finally, we have to take into consideration medications used in JIA as they may also adversely affect a patient’s cardiac condition [37]. For example, chloroquine has cardio-toxic side effects [38] while, Sulphasalazine, Methotrexate, Nonsteroidal Anti-inflammatory Drugs and Steroids do not have known direct cardio-toxic side effects. However, prolonged use of steroids predisposes the patients to increased risk of hypertension [39] and cardiac events [40]. Medications used by our patients were described in Table 1, where most of them were treated with Methotrexate (MTX). Only four patients were on low dose steroid (<10 mg/day) and nine patients had a past history of steroid intake either low dose oral steroids or local joints steroid injections, ensuring that our findings are not related to medications side effects.

## Conclusion

In conclusion, intra- and interatrial electromechanical delays are prolonged and LA mechanical functions are impaired in active JIA patients. So, it may be an early form of subclinical cardiac involvement in JIA patients with no clinical evidence for cardiovascular affection. Disease duration and disease activity are independent factors of the intra-atrial electromechanical delay. In addition, our results, suggests that significant diastolic functional abnormalities exist in JIA children.

### Clinical implications

Cardiac evaluation for JIA patient should be included in the routine follow for those patients especially blood pressure monitoring. TDI is considered a valuable method for assessment of subclinical cardiac involvement.

### Study limitations

We acknowledge some limitations in the present study. First small sample size and selection bias of cases being all active JIA which hampers the generalization of the results. Second, the cross sectional study gives a potential association between the exposure and the outcome, not causation so longitudinal analyses (cohort study) would be reasonable to assess the long-term effect of the disease on the heart which is essential in summing up a final conclusion. Third, there are some unmeasured confounders as socioeconomic status and adherence to treatment.

## Abbreviations

A: Peak atrial filling velocity
ACR: American College of Rheumatology
AEMD: Atrial electromechanical delay
AF: Atrial fibrillation
Am: Late diastolic velocity
Am wave: Late diastolic wave
BMI: Body mass index
BSA: Body surface area
CRP: C reactive protein
E: Peak early
E/A: Ratio of E to A
ECG: Electrocardiography
Edt: E deceleration time
EF: Ejection fraction
Em: Early diastolic velocity
ESR: Erythrocyte sedimentation rate
FS: Fractional shortening
ILAR: International League of Associations for Rheumatology
IVRT: Isovolumic relaxation time
JADAS −27: Juvenile arthritis disease activity score
JIA: Juvenile idiopathic arthritis
LA: Left atrial
LV: Left ventricular
LVEDD: Left ventricular end diastolic diameter
LVESD: Left ventricular end systolic diameter
PA lateral: Lateral mitral annulus
PA septum: Septal mitral annulus
PA tricuspid: Right ventricular tricuspid annulus
PWD: Pulse Wave Doppler
RF -ve: Negative rheumatoid factor
Sm: Systolic velocity
TDI: Tissue Doppler imaging
TDI: Tissue Doppler imaging
TTE: Transthoracic echocardiographic
VAS: Visual analog scale
